# A retrospective 10 years‐ experience overview of dye laser treatments for vascular pathologies

**DOI:** 10.1111/srt.13427

**Published:** 2023-08-02

**Authors:** Cannarozzo Giovanni, Pennati Beatrice Marina, Zingoni Tiziano

**Affiliations:** ^1^ Lasers in Dermatology Unit Università Roma Tor Vergata Rome Italy; ^2^ Clinical Research and Practice Department El.En. Group Calenzano Italy

**Keywords:** cherry angioma, dye laser, infantile haemangioma, port wine stain, rhinophyma, spider angioma, telangiectasia, vascular pathologies, vascular tumours

## Abstract

**Introduction:**

The Flash‐lamp pulsed dye laser (FPDL) is nowadays considered the most precise laser currently on the market for treating superficial vascular lesions. In this study, we gathered data from 10 years of experience regarding dye laser treatment of patients presenting vascular malformations such as telangiectasia, rhinophyma, port‐wine stain, cherry and spider angioma and vascular tumours.

**Methods:**

Subjects were enrolled from 2013 to 2023 based on the vascular anomalies they presented. They underwent different treatment sessions with the FPDL device.

**Results:**

The age‐range distribution by vascular anomaly confirmed that haemangiomas are typical in children while rhinophyma is a condition very common in older adults. A difference in sex distribution showed that pathologies such as telangiectasias typically affect women whereas rhinophyma is more frequent in men. Most of the treatments interested the face area but no permanent side effects were registered.

**Conclusions:**

Our 10 years of experience with FPDL demonstrated good results in a wide range of applications for the treatment of different vascular anomalies. The absence of long‐term side effects and bearable pain during the treatment makes it a valuable solution for the resolution of benign tumours also in very young patients.

## 1 INTRODUCTION

The Flash‐lamp pulsed dye laser (FPDL) is nowadays considered the most precise laser currently on the market for treating superficial vascular lesions. It uses a rhodamine dye mixed in a solvent and pumped by a flash lamp to produce an emission at a wavelength of 595 nm, which is roughly close to the absorption peaks of haemoglobin and oxyhaemoglobin.^1^ The cornerstone theory behind FPDL is the concept of selective photothermolysis, which targets specific skin structures for damage while sparing nearby tissue and relies on as‐yet‐unexplored mechanisms (such as immune‐modulating mechanisms). FPDL emits visible light pulses with a wavelength of 585 or 595 nm. However, hyperpigmentation is a potential negative side effect.^2^ The FPDL utilising the wavelength of 585 nm can target the capillaries, therefore the coagulation should inhibit the replication of warts and accelerate their recovery. The FPDL works by selectively photothermolyzing cutaneous blood vessels without affecting the surrounding tissue, which lowers the risk of scarring.^3^ The destruction of the virus is aided by photothermolysis of cutaneous blood vessels because it increases the production of inflammatory cytokines and cellular immunological responses. Additionally, the wart's recovery is aided by immunomodulating qualities.^3^

It is believed to be the gold standard treatment for many vascular problems for all these reasons. According to the International Society for the Study of Vascular Anomalies (ISSVA) classification, vascular anomalies could be divided into vascular tumours (caused by vascular endothelial cell proliferation) and vascular malformations (structural abnormalities). Within the first group, there are benign (e.g., congenital, or infantile haemangioma), locally aggressive or borderline (e.g., Kaposi's sarcoma and kaposiform haemangioendothelioma) and malignant (e.g., epithelioid haemangioendothelioma and angiosarcoma) formations. On the contrary, vascular malformations are divided into four types. The Simple type includes capillary, lymphatic, venous, and arteriovenous malformations. A particular lesion may have two or more simple vascular abnormalities in a combined type. The term ‘major named vessels malformation’ describes anomalies in the origin, course or quantity of major blood vessels with anatomical names. Symptoms other than vascular anomalies, such as soft tissue or skeletal abnormalities, such as leg‐length discrepancy and segmental hypertrophy, can complicate vascular malformations in syndromes (such as Klippel‐Trenaunay syndrome and Sturge‐Weber syndrome).^4^, ^5^

**FIGURE 1:**
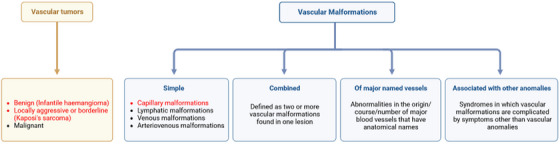
Overview of the ISSVA classification for vascular anomalies (modified from^4^). In red, are those that could be treated with FPDL.

Among these lesions, FPDL had been demonstrated to be effective in the treatment of capillary malformations especially when localised on the face. Moreover, it showed good results for the resolution of benign, and borderline vascular tumours such as infantile haemangiomas and Kaposi's sarcoma (see Figure 1).^6^, ^7^

Laser therapy can be performed using a variety of lasers, including pulsed dye lasers (PDL), carbon dioxide (CO2) lasers and neodymium‐doped yttrium aluminium garnet (Nd:YAG) lasers. These devices were typically suggested as an alternative when all other nonsurgical local therapies failed.^8^, ^9^, ^10^ Moreover, FPDL is often used in combination with other treatments for treating vascular‐dependent lesions. Lasers used to treat vascular lesions include FPDL, Nd:YAG lasers, intense pulsed light devices and CO2 lasers.

### 1.1 Infantile haemangioma laser therapy

Placental tissue may be the source of the proliferating embryonal tumours known as infantile haemangiomas (IHs). A fascinating hypothesis reported by Bruscino et al.^11^ contends that haemangiomas develop from embolised placental cells inside the uterus which is the ideal environment for these cells to live and grow. It is the most prevalent benign tumour in infancy and in Caucasian newborns, the prevalence of haemangiomas ranges from 10 to 12%, although it is less common in African and Asian children. Most of these vascular lesions (50%−60%) are superficial.

In our experience, the laser treatment of this vascular anomaly represents a preventive strategy for scar formation. Indeed, even if about 90% of IHs naturally regress, partially or entirely, within the 7th year of life (usually between the 3rd and the 6th year)^12^, ^13^, ^14^ in 10–15% of cases, treatment is required. In detail, in 50% of cases, the tumour regresses without any clinical evidence but in the other 50% of cases, scarring, telangiectasias, wrinkling and dyspigmentations may be present. Specifically, drug‐based treatments with oral propranolol have been largely proven to be the first‐choice drug for IH in paediatric patients.^15^ The medication has an ideal safety profile and is extremely effective (up to 96%–98% after 6 months). As specified by the drug's summary of product characteristics (SmPC—European Medicines Agency),^16^ infants between the ages of 5 weeks and 5 months should receive oral propranolol for a period less than 6 months.^17^ For all those children who are not treated in this window time, no medication has now satisfactory results. That is why 585 or 595 nm laser treatments could be used to prevent scarring which is usually a reason for a social and psychological impact on a child's life. Indeed, when haemangiomas occur in high‐risk areas, such as near the eyes, throat, nose, or trunk (60%) possibly impairing their function, it is important for an accurate medical intervention.^17^

In this study, we gathered data from 10 years of experience regarding dye laser treatment of patients presenting vascular malformations such as telangiectasia, rhinophyma, port‐wine stain (PWS), cherry angioma (ruby angioma), spider angioma and vascular tumours like IH. With this overview, we wanted to highlight the advantages of flash‐lamp pulsed dye lasers.

## 2 MATERIALS AND METHODS

### 2.1 Enrolled population

For this scientific research, the subjects were enrolled from 2013 to 2023 based on the vascular anomalies they presented. Details are shown in Table 1 and Figure 3.

**TABLE 1:** The number of patients and their characteristics by type of vascular anomaly are reported.

Vascular anomaly	Phototype range	Body area
Telangiectasia (resistant to Nd:YAG 1064 nm laser treatment)	II–III	Face
Rhinophyma (for vascular reduction)	II–III	Nose
Port‐wine stain (PWS)	II–III	Face 40% Neck 30% Trunk 20% Arms 10%
Haemangioma (scar prevention after spontaneous vascular regression or in patients who have not received b‐blocker therapy within the window‐time administration)	II–III	Face (max diameter = 5 cm)
Cherry Angioma (ruby angioma)	II–III	Trunk 70% Legs 30 %
Spider Angioma	II–III	Face

The patients were clinically examined, and anamnestic data were collected to select the most appropriate treatment. General information such as the type of lesion to be treated (size, depth, location, colour, severity), phototype, age, family history, motivations and expectations, and recent sun exposure was obtained. The patients were extensively informed on the treatment procedure and details and informed consent was obtained. Patients taking photosensitising drugs, anticoagulants, retinoids (which could alter the normal tissue repair), had previous exfoliating treatments (peeling, scrubs, retinoic acid etc.) or surgical treatments (lifting etc.), exposed to the sun or ultraviolet lamps (before, during and after the treatment), or have bacterial, viral or mycotic infections in progress, were contraindicated for the study. Specifically, exclusion criteria regarded any hypersensitivity to the light, pregnancy, or previous skin cancer or the presence of pre‐cancerous lesions.

### 2.2 Clinical assessment

At the baseline and after the last treatment, high‐resolution digital photographs of the treated area were taken.

### 2.3 Multispectral assessment

An optical evaluation of the skin structures to monitor the effects of treatment was performed using an in vivo multispectral skin imaging with Antera 3D (Antera 3D; Miravex Limited, Dublin, Ireland). This tool uses a computer‐assisted reconstruction of skin surfaces and multidirectional lighting to assess the melanin and vascular components.

### 2.4 Workflow

Before treatment the interested area was cleaned removing all impurities that could interact with the light radiation (make‐up, lotions, deodorants, ointments etc.) and patients were advised not to use cosmetics for 48 h prior to treatment as a precaution.

A skin test was performed before the beginning of the treatment to establish the proper therapy for every patient. Topical anaesthesia was not required. Indeed, patients usually well tolerated the treatment thanks to the skin cooling systems used before, during, and after the procedure.

Normally, the procedure foresees passing the handpiece on the skin's surface with overlapping areas up to 20%. When treating areas close to bone surfaces (forehead, cheekbones, etc.), special caution was used because these areas reflect the laser light, increasing the amount of energy dissipated. To do so, the fluence was reduced as much as necessary. Wads of cotton wool soaked in fresh water should be used to cover the teeth when treating the peri‐labial region or the cheeks.

Depending on the region, energy fluence, skin type, and pulse duration, PDL therapy could cause erythema, oedema, purpura, crusting, blistering, hyper‐ or hypopigmentation, and in rare cases, scarring.

Potential adverse events include post‐inflammatory pigmentary changes (especially in darker‐skinned patients), immediate post‐laser purpura, recurrence, and infection. Surface cooling has markedly diminished this side effect. Swelling and erythema are frequently present immediately after treatment, especially around the eyes, but resolve within 24–48 h.

### 2.5 Device and protocols

Patients underwent different treatment sessions with the FPDL device (Synchro VasQ, DEKA, Florence, Italy) as indicated by Table 2. The device was provided with a skin contact sensor to assure the best results. A jet of air as the cooling system (Cryo6, Zimmer) was applied to the treated area to reduce oedema and inflammation during the treatment.

**TABLE 2:** Protocol details by vascular anomaly. The number of sessions and the interval they were performed, the device settings, the endpoint and the common side effects are reported.

Treatments	Sessions plan	Settings	End‐point	Common side effects
Telangiectasia (resistant to Nd:YAG 1064 nm laser tx)	1–2 Tx every 40 d	5÷12 mm Spot, 6.5÷9 J/cm^2^, 0.5÷3 ms	Slight purpura and oedema	Transient hyperpigmentation
Rhinophyma (for vascular reduction)	1–2 Tx every 40 d	10÷12 mm Spot, 6.5÷9 J/cm^2^, 0.5÷1.5 ms	Slight purpura and oedema	Transient purpura (disappears in 10/12 d) Subepidermal blister Transient hyperpigmentation (disappears in 60–90 d, very rare)
Port‐wine stain (PWS)	6–9 Tx every 90–120 d	10÷12 mm Spot, 6.5÷9 J/cm^2^, 0.5÷1.5 ms	Slight purpura and oedema	Transient purpura (disappears in 10/12 d) Subepidermal blister Transient hyperpigmentation (disappears in 60–90 d) Keloids (very rare): can be re‐treated with Dye laser
Haemangioma (scar prevention after spontaneous vascular regression or in patients who have not received b‐blocker therapy within the window‐time administration)	8–10 Tx every 60–90 d	7÷12 mm Spot, 6÷8.5 J/cm^2^, 0.5÷3 ms	Slight purpura and oedema	Transient purpura (disappears in 10/12 d) Subepidermal blister Transient hyperpigmentation (disappears in 60–90 d)
Cherry Angioma (ruby angioma)	1–2 Tx every 30 d	7÷12 mm Spot, 5.5÷8 J/cm^2^, 0.5÷3 ms	Slight purpura and oedema	Transient hypo or hyperpigmentation
Spider Angioma	1–2 Tx every 30 d	5÷12 mm Spot, 7÷9 J/cm^2^, 0.5÷1.5 ms	Slight purpura and oedema	Transient hypo or hyperpigmentation

Abbreviation: d = days.

*Tx = treatment.

### 2.6 Post‐treatment care

After 4/5 days from the end of the treatment, patients were visited for a control. Right after the last session, a slight compression of the skin with cold wet gauzes or cooled by a jet of air from the cooling system (Cryo6, Zimmer) was applied to the treated area to reduce oedema and inflammation. Moreover, without massaging, a non‐cortisone anti‐inflammatory cream or unguent was applied (zinc oxide based). Patients were advised not to use hot water on the treated area for 24 h, play sports (swimming especially) for 48 h, rub the treated area, and be exposed to direct sunlight.

## 3 RESULTS

**FIGURE 2:**
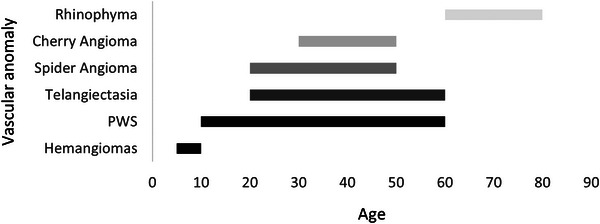
Vascular anomalies divided by patients’ age. There are pathologies typically affecting specific age ranges (e.g., haemangiomas in children and rhinophyma in people in their 60s).

The general characteristics of the study population were analysed. In Figure 2 is possible to see the age‐range distribution by vascular anomaly. In general, haemangiomas are typical in children (≤10 years old) while rhinophyma is a condition very common in older adults (≥60 years old).

**FIGURE 3:**
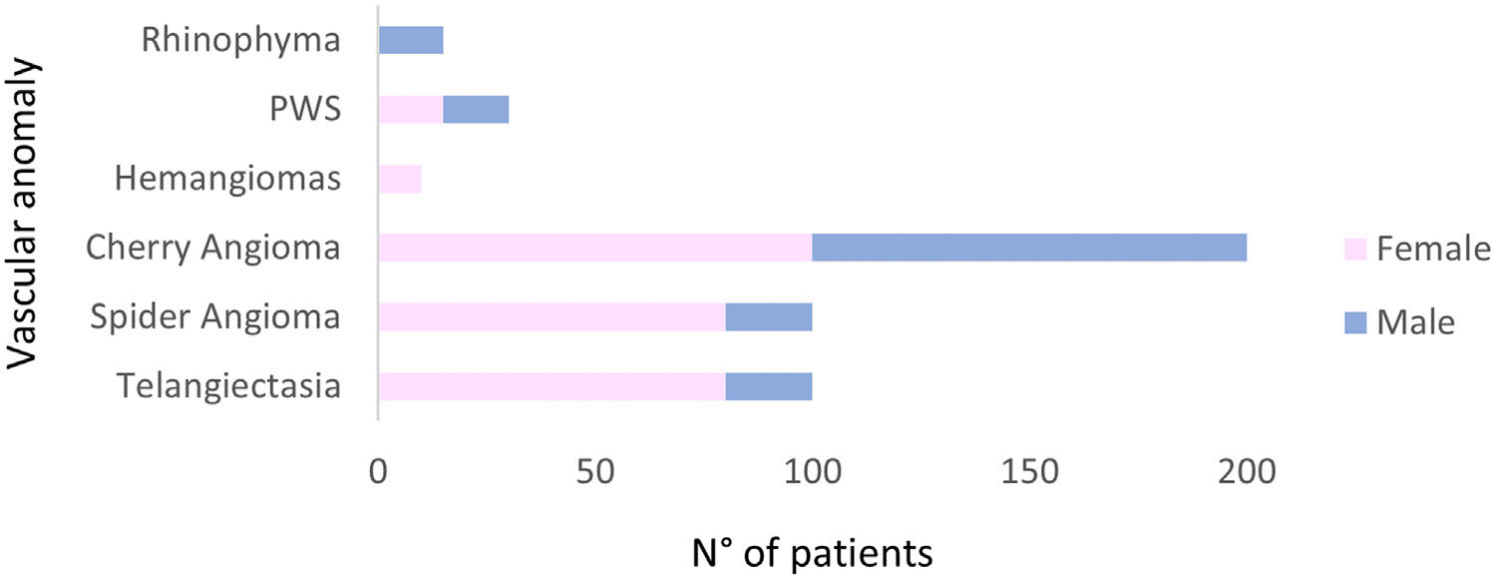
Vascular anomalies divided by patients’ sex. There are pathologies typically affecting women (e.g. haemangiomas in children, telangiectasia) or men (e.g., rhinophyma).

Moreover, is possible to appreciate a difference in sex distribution by vascular anomaly. So, there are pathologies such as telangiectasias that typically affect women. And, on the contrary, rhinophyma is more frequent in men (see Figure 3).

**FIGURE 4:**
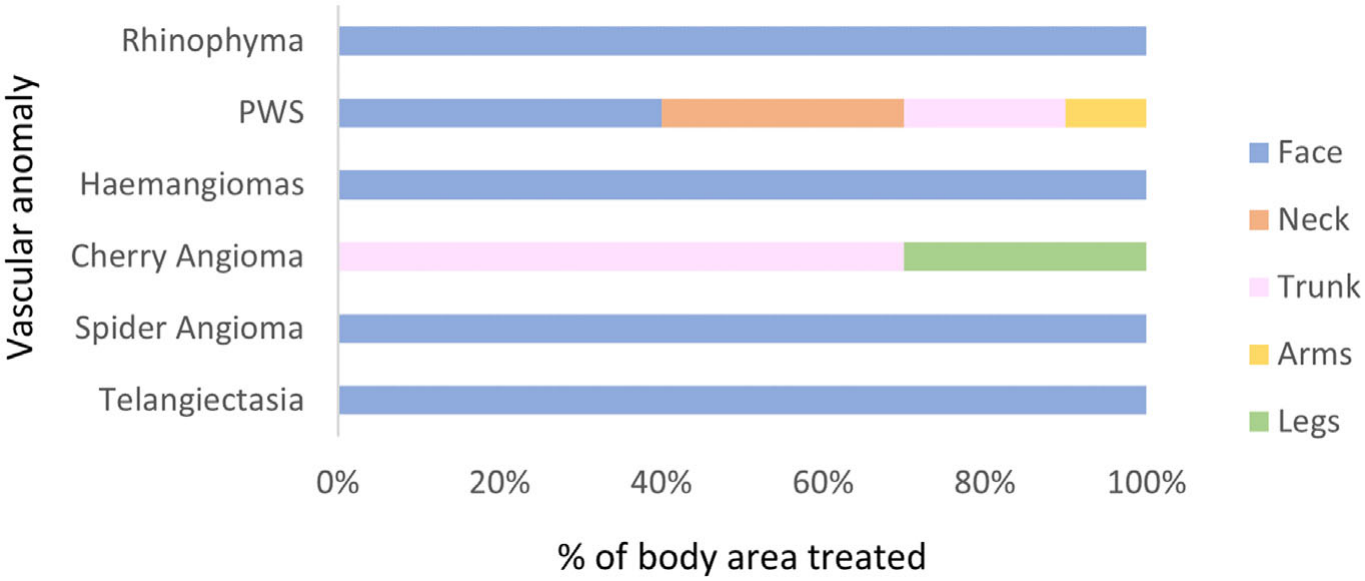
Vascular anomalies divided by body area affected. There are pathologies typical of the face area (e.g., spider angiomas, telangiectasias) and others most common on the trunk and limbs (e.g., port wine stains, cherry angiomas).

Most of the treatments interested the face area as shown in Figure 4. The exceptions are represented by the cases of cherry angioma and PWS that typically affect the limbs and the trunk.

**FIGURE 5:**
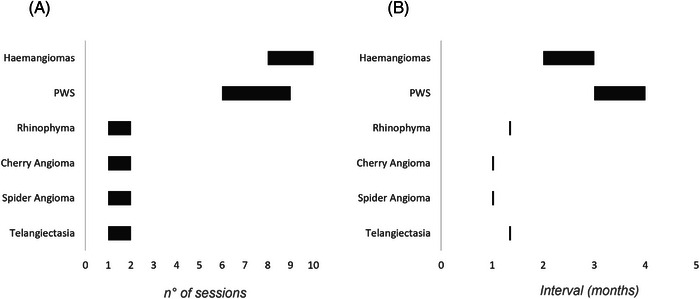
Vascular anomalies divided by the number of treatment sessions (A) and by the intervals (in months) between the treatment sessions (B).

Different pathology is a synonym of different protocols and procedures to be followed and so the number of sessions to be performed. Figure 5 clearly shows how, for example, haemangiomas and port‐wine stains require many treatment sessions before having a visible and acceptable result. Moreover, these two pathologies require a longer interval between the treatment sessions to have a better result and minimal side effects.

### 3.1 Pre‐ and post‐treatment assessment

**FIGURE 6:**
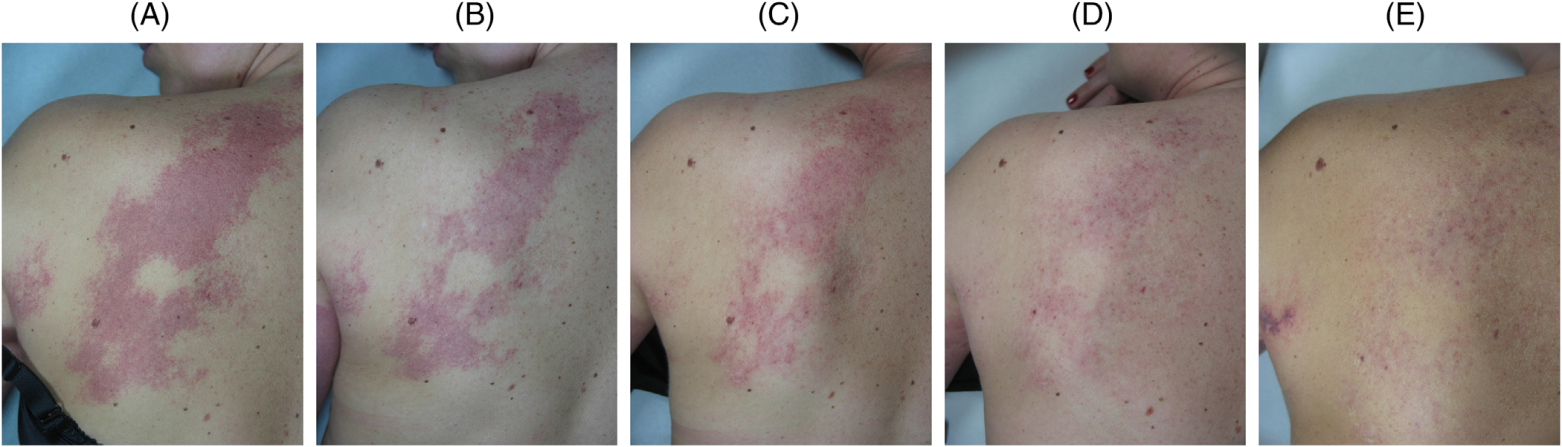
Clinical assessment of a shoulder port wine stain in a female patient. The progression from A to E shows an almost full resolution. A = before treatment; B = after one treatment; C = after two treatments; D = after four treatments; E = after six treatments.

**FIGURE 7:**
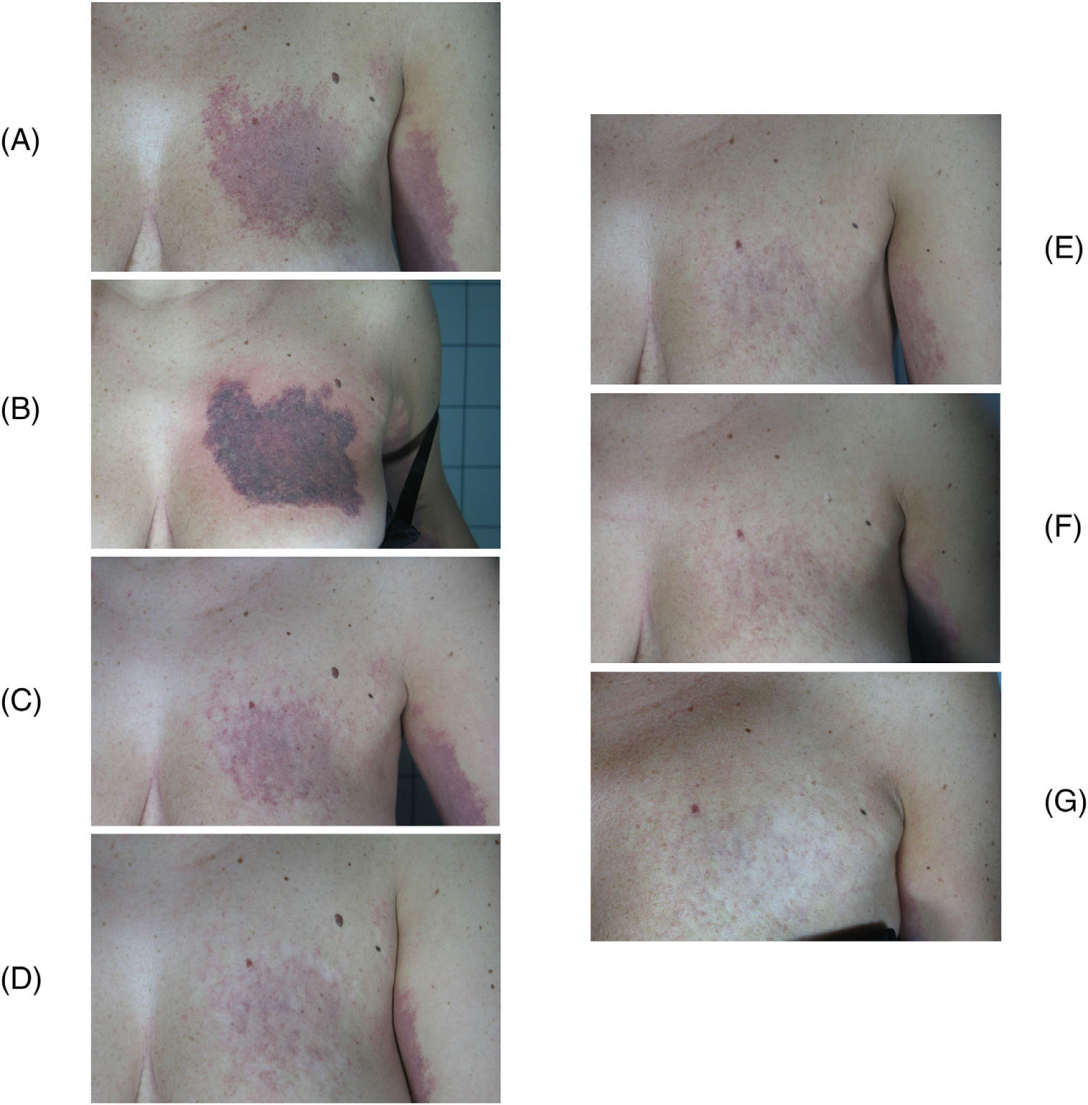
Clinical assessment of a breast port wine stain in a female patient. The progression from A to G shows an almost full resolution. A = before treatment; B = right after treatment (the endpoint with purpura is visible); C = after one treatment; D = after two treatments; E = after three treatments; F = after four treatments; G = after six treatments.

**FIGURE 8:**
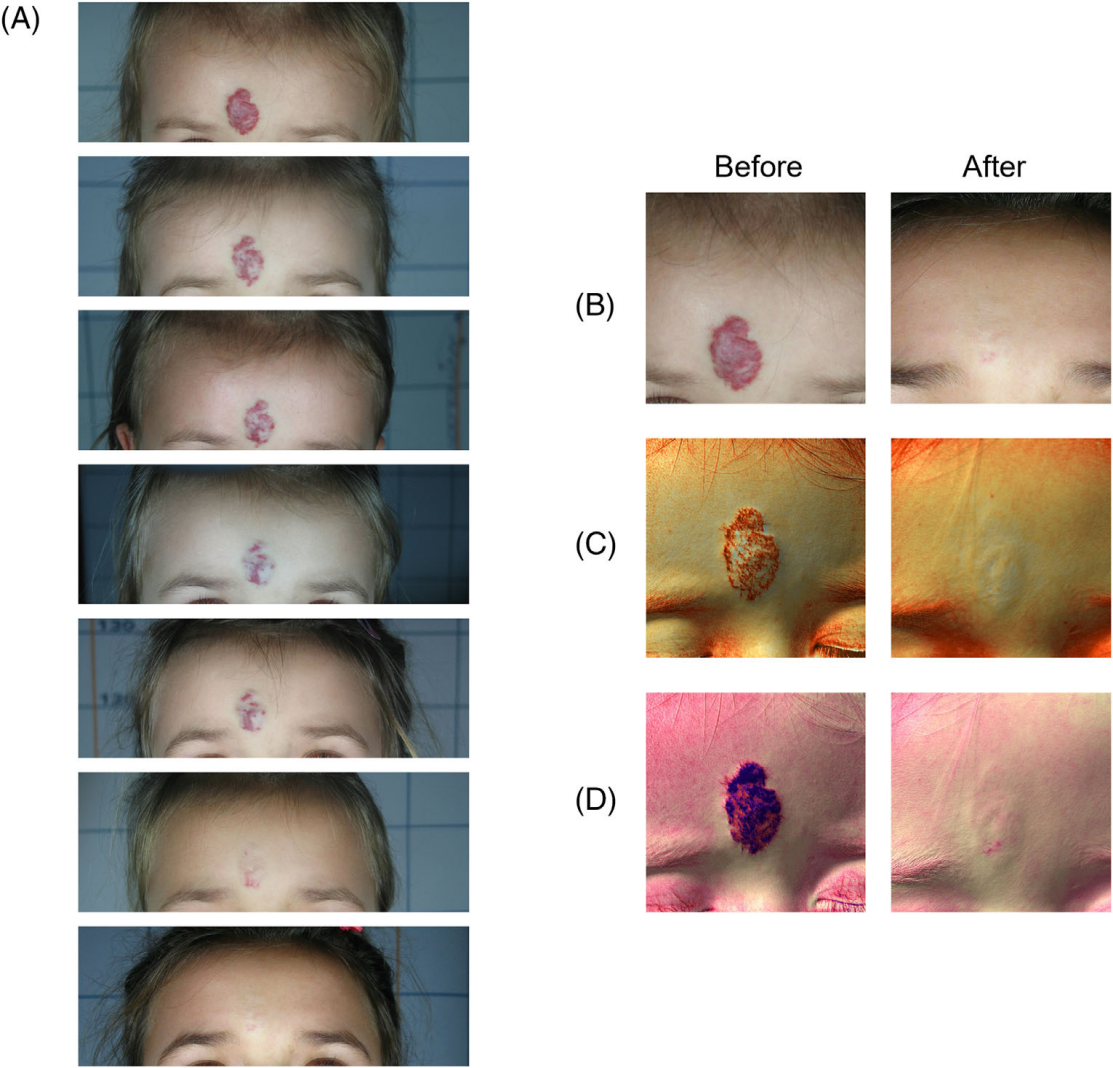
Clinical (A and B) and multispectral (G and H) assessment of a male patient presenting a port wine stain in the superior lip area. The melanin (G) and vascular (H) components are highlighted with Antera 3D (Antera 3D; Miravex Limited, Dublin, Ireland) system before and after the last treatment. An almost full resolution of the vascular anomaly is visible with and without image filters. A = before treatment; B = right after treatment (the endpoint with purpura is visible); C = after one treatment; D = after three treatments; E = after six treatments; F = clinical assessment before and after the last treatment; G = multispectral melanin component, before and after the last treatment; H = multispectral vascular component, before and after the last treatment.

**FIGURE 9:**
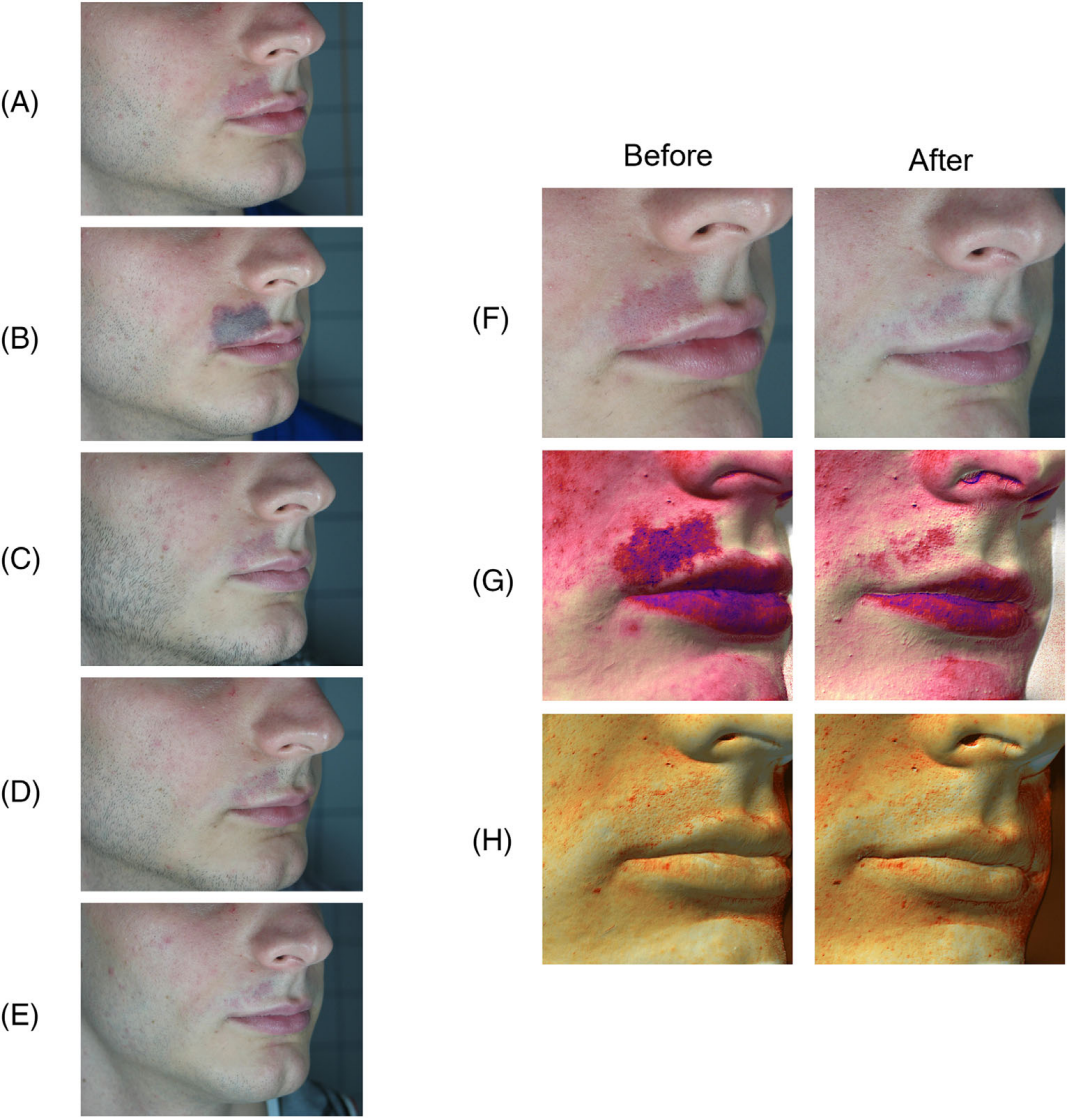
Clinical (A and B) and Multispectral (C and D) assessment of a child presenting an infantile haemangioma in the forehead. The melanin (C) and vascular (D) components are highlighted with the Antera 3D (Antera 3D; Miravex Limited, Dublin, Ireland) system. A full resolution of the vascular anomaly is visible with and without image filters. B = clinical assessment before and after the last treatment; C = multispectral melanin component, before and after the last treatment; D = multispectral vascular component, before and after the last treatment.

**FIGURE 10:**
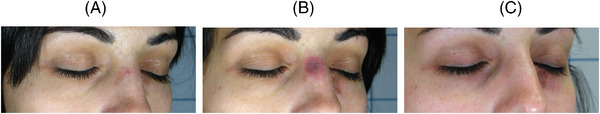
Clinical assessment of a nose spider angioma in a woman patient. The progression from A to C shows an almost full resolution. A = before treatment; B = right after treatment (the endpoint with purpura is visible); C = after one treatment.

**FIGURE 11:**
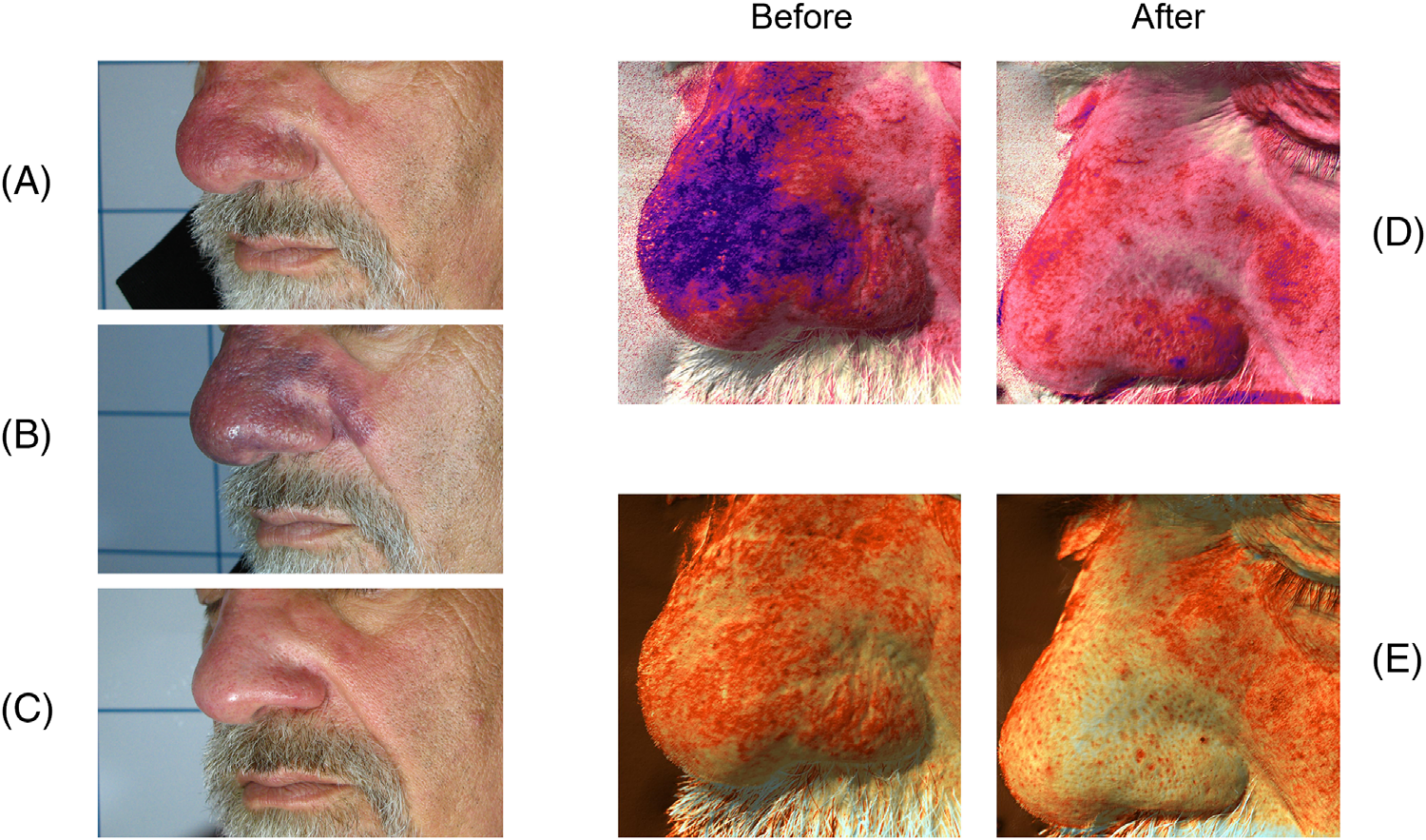
Clinical assessment of trunk cherry (ruby) angiomas (A) in a woman patient. The progression and a full resolution are visible also when the dermatoscopic analysis is performed (B) before (1), right after (2), and after (3) the last treatment (just 1).

**FIGURE 12:**
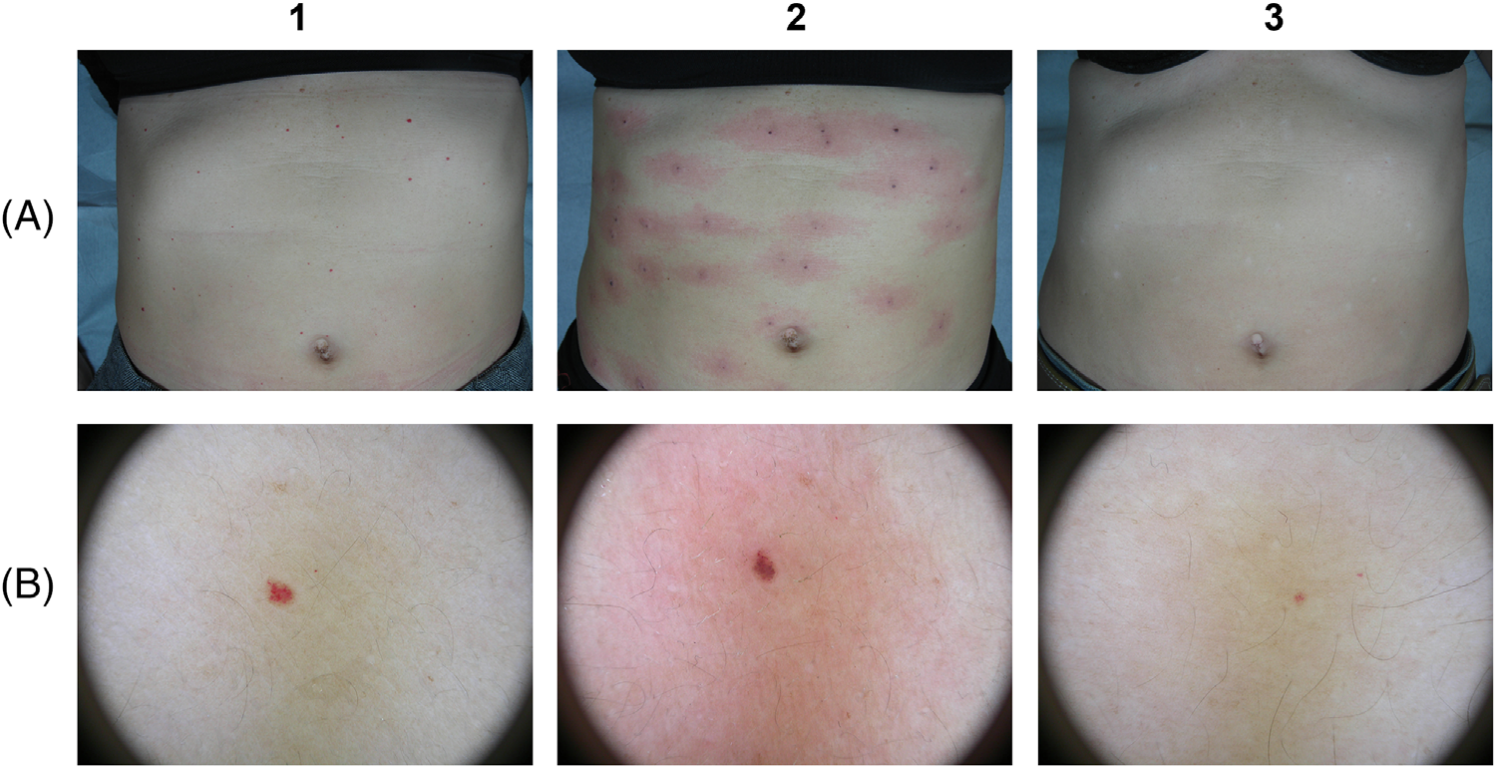
Clinical (A) and multispectral (D and E) assessment of a male patient presenting rhinophyma. The melanin (D) and vascular (E) components are highlighted with Antera 3D (Antera 3D; Miravex Limited, Dublin, Ireland) system before and after the last treatment. A full resolution of the vascular anomaly is visible with and without image filters. A = before treatment; B = right after treatment (the endpoint with purpura is visible); C = after two treatments; D = multispectral melanin component, before and after the last treatment; E = multispectral vascular component, before and after the last treatment.

The clinical and multispectral assessments were carried out for every patient at the baseline, during the process and after the last session. We here show some exemplificative clinical cases. Figures 6, 7 and 8 report port wine stain case progresses after treatment with FPDL. An almost full resolution is visible with no permanent side effects. Figure 9 shows an IH while Figures 10 and 11 report spider and cherry angiomas, respectively. Lastly, Figure 12 is a rhinophyma resolution.

## 4 DISCUSSION

Simple vascular malformations therapy typically makes use of the intense flash‐lamp pulsed dye laser. Similar to earlier research,^1^, ^7^ various vascular lesions were treated in this investigation solely utilising the FPDL laser. When other treatments are not indicated by contraindications or for resistant and/or refractory lesions, FPDL may be used as an alternative or complementary therapy. Our experience confirms that is safe, well‐tolerated, and successful in all cases. Moreover, it enables to treat efficiently patients with benign vascular tumours. They were paediatric subjects with face haemangiomas with no results after the canonical pharmacological with β‐blockers treatment or that cannot have had the treatment in the correct window time. Our data evidenced an overall patient improvement, but it remains crucial to correctly select in advance the patients to be treated with FPDL. Indeed, this technique is beneficial in patients with vessel‐related chronic and/or hard‐to‐resolve dermatological conditions but also with the right aptitude to accept and understand the treatment procedure. For instance, children and their parents often are fearful about the treatment and prefer to avoid uncomfortable procedures.^18^ On the contrary, FPDL is particularly suggested in patients using anticoagulants, those with cardiopathies, and those who cannot obtain anaesthesia or have difficulties undergoing surgery. Also, FPDL is advised in regions (neck, face, nasal wings, nose, groynes and anogenital areas) where surgical devices can possibly disfigure scars or result in keloids.

The majority of the lesions treated had a significant number of dilated blood vessels, which serve as the device's target, and contribute to the efficacy of FPDL treatment. The mechanism of action of FPDL is thus based on the destruction of targeted abnormal vessels, elements of the lesions themselves, or a selective vessel thrombosis with the resulting elimination of the nutrient supply to the lesions. Additionally, researchers have found that the FPDL can reduce collagen type III deposition and fibroblast proliferation, as well as possibly trigger apoptosis and an increase in p38 mitogen‐activated protein kinase and extracellular signal‐regulated kinase activity. Therefore, its use in other dermatological disorders like keloids is possible.^1^, ^19^, ^20^

Nowadays, the optimization of laser devices, parameters and settings has led to the employment of higher fluences for targeting deeper structures.^21^ As Bruscino et al.^11^ reported, the choice of longer pulse widths and wavelengths and the use of cooling systems have permitted dermatologists to achieve faster results.^22^ Even if this choice is still effective and with few side effects, we decided to further minimize the unwanted aspects of the procedure reducing the fluences and enlarging the spot sizes. Indeed, none of the patients we treated showed any discomfort or permanent side effect after the treatment sessions.

Years of clinical expertise have enabled continuous optimising of standard parameters for known treatments and finding new therapeutic horizons. With diverse and occasionally less well‐known indications FPDL can produce very good effects. FPDL is now only valued as an alternate, additional or experimental treatment for other reasons. FPDL's numerous new modifications and techniques will lead to both an expansion of the treatment's indications and a further improvement in therapeutic efficacy.^23^

## 5 CONCLUSION

Our 10 years of experience with FPDL demonstrated good results in a wide range of applications for the treatment of different vascular anomalies. The absence of long‐term side effects and bearable pain during the treatment makes it a valuable solution for the resolution of benign tumours also in very young patients.

### 5.1 Study limitations and future perspectives

Despite the good aesthetic results, the procedure's high price may put a limit on its utilisation.

## CONFLICT OF INTEREST STATEMENT

BMP and TZ are employed at El.En. Group. The other authors declare that the research was conducted in the absence of any commercial or financial relationships that could be construed as a potential conflict of interest.

## ETHICS STATEMENT

The study was conducted in accordance with the Declaration of Helsinki. As the device has been an already CE‐marked device since 2019, ethical review and approval were waived for this study. Informed consent was obtained from all subjects involved in the study.

## Data Availability

The data that support the findings of this study are available upon request from the corresponding author. The data are not publicly available due to privacy or ethical restrictions.
