# Correction to A retrospective 10 years‐of‐experience overview of dye laser treatments for vascular pathologies

**DOI:** 10.1111/srt.13458

**Published:** 2023-09-05

Giovanni, C, Marina, PB, Tiziano, Z. A retrospective 10 years‐ experience overview of dye laser treatments for vascular pathologies. *Skin Res Technol*. 2023; 29:e13427. https://doi.org/10.1111/srt.13427

Figures 8 and 9 are inverted, and so are figures 11 and 12. The correct figures and captions are shown below.

We apologize for this error.

**FIGURE 8:**
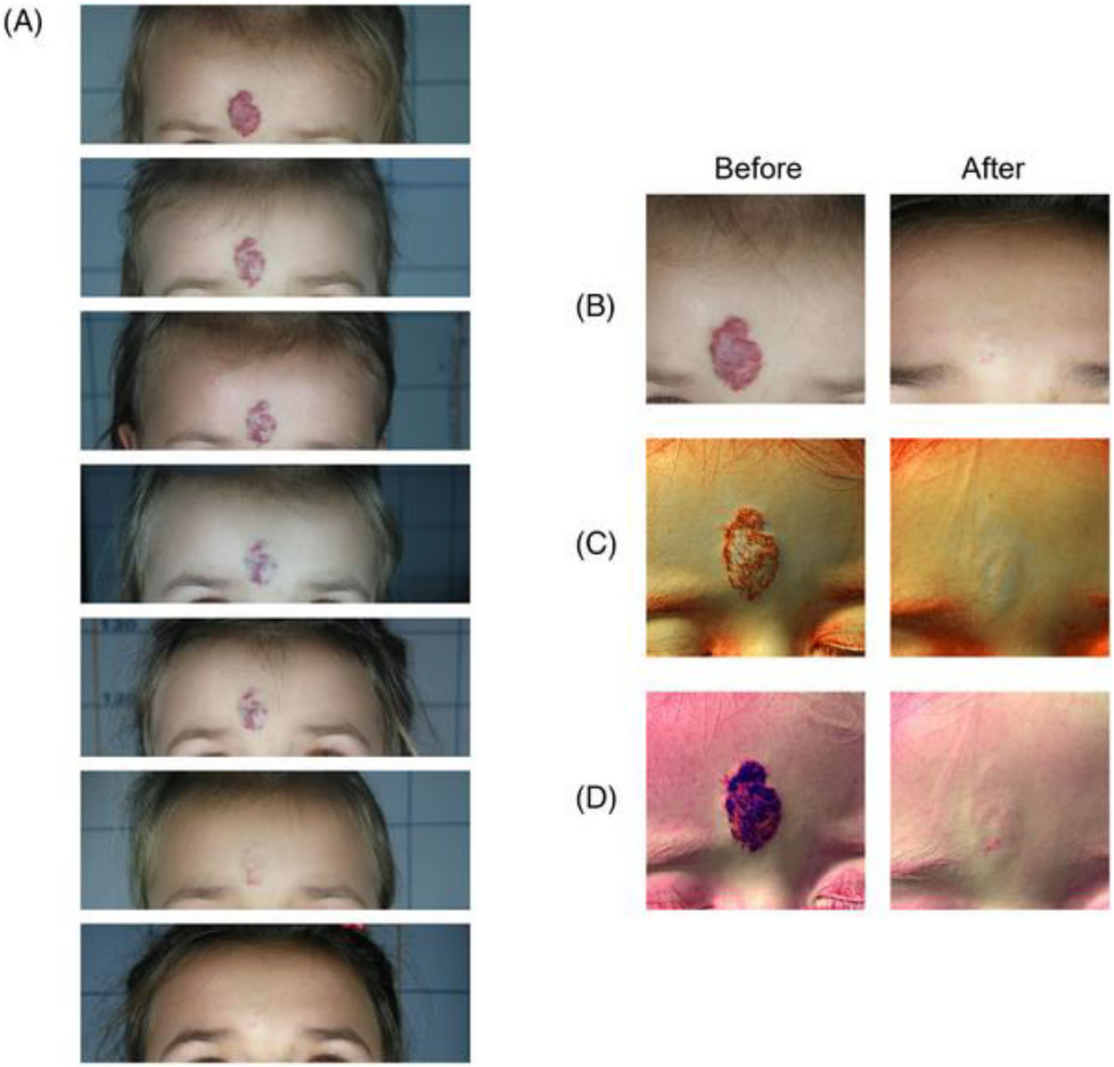
Clinical (A and B) and Multispectral (C and D) assessment of a child presenting an infantile haemangioma in the forehead. The melanin (C) and vascular (D) components are highlighted with the Antera 3D® (Antera 3D; Miravex Limited, Dublin, Ireland) system. A full resolution of the vascular anomaly is visible with and without image filters. B = clinical assessment before and after the last treatment; C = multispectral melanin component, before and after the last treatment; D = multispectral vascular component, before and after the last treatment.

**FIGURE 9:**
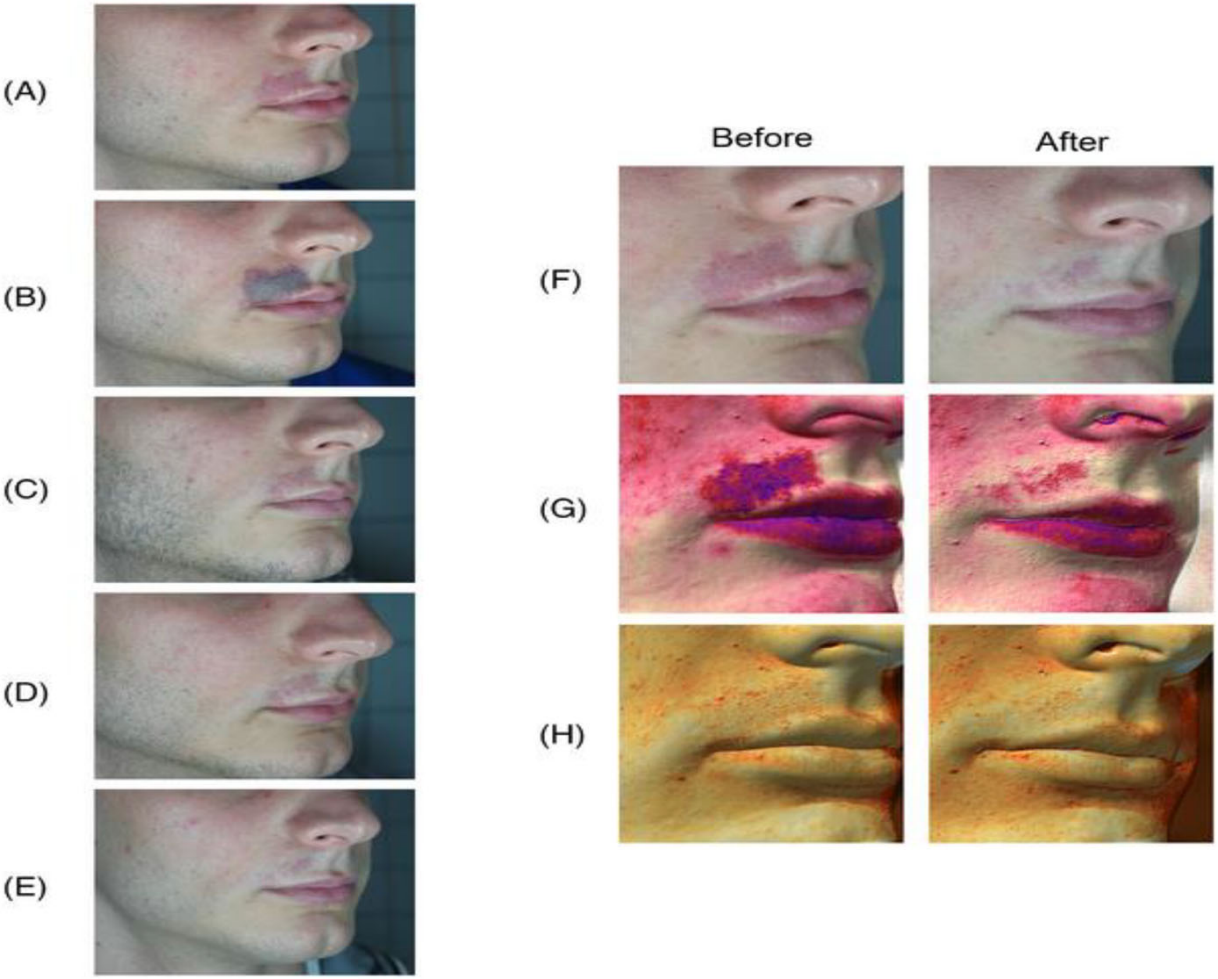
Clinical (A and B) and Multispectral (G and H) assessment of a male patient presenting a port wine stain in the superior lip area. The melanin (G) and vascular (H) components are highlighted with Antera 3D® (Antera 3D; Miravex Limited, Dublin, Ireland) system before and after the last treatment. An almost full resolution of the vascular anomaly is visible with and without image filters. A = before treatment; B = right after treatment (the endpoint with purpura is visible); C = after 1 treatment; D = after 3 treatments; E = after 6 treatments; F = clinical assessment before and after the last treatment; G = multispectral melanin component, before and after the last treatment; H = multispectral vascular component, before and after the last treatment.

**FIGURE 11:**
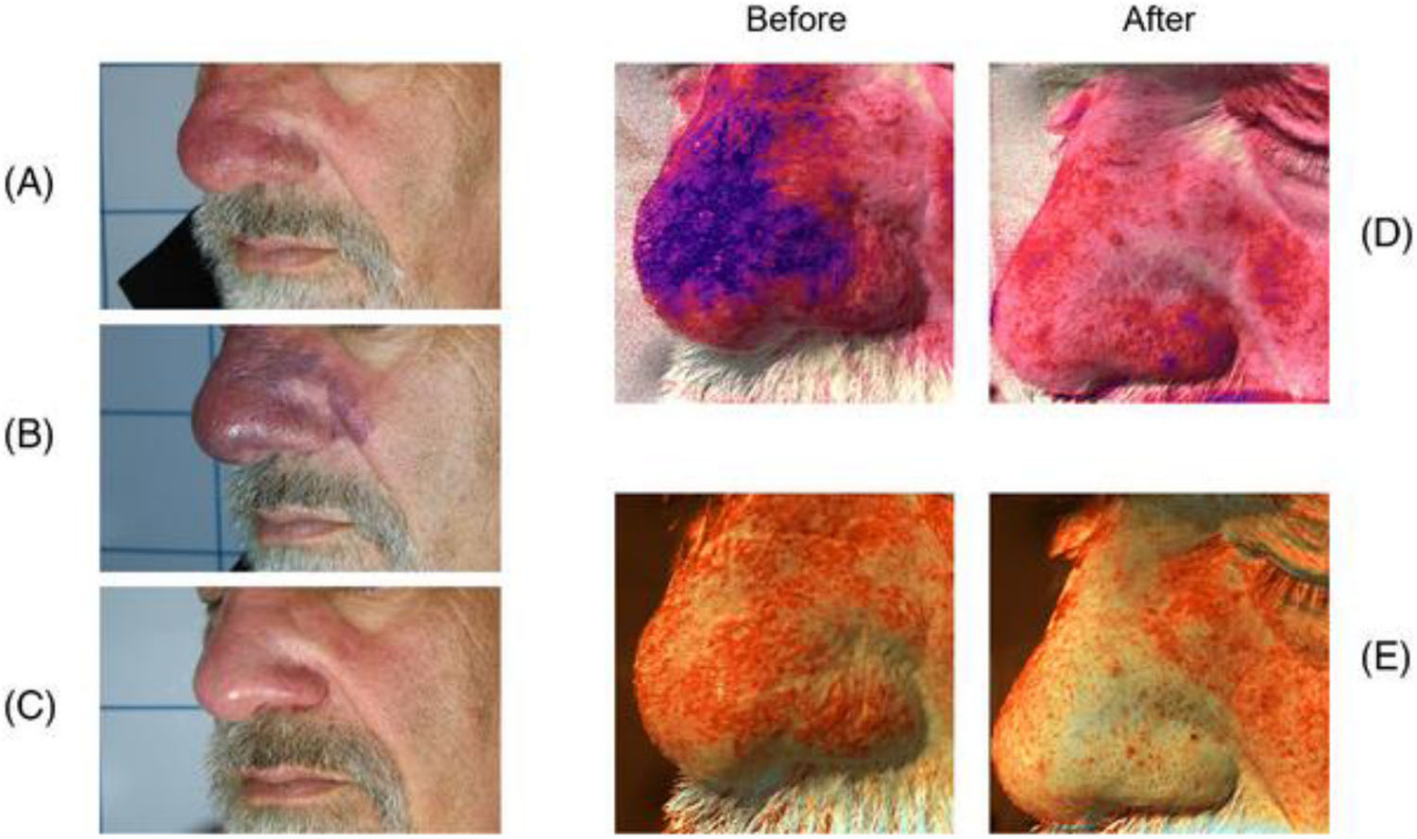
Clinical (A) and Multispectral (D and E) assessment of a male patient presenting rhinophyma. The melanin (D) and vascular (E) components are highlighted with Antera 3D® (Antera 3D; Miravex Limited, Dublin, Ireland) system before and after the last treatment. A full resolution of the vascular anomaly is visible with and without image filters. A = before treatment; B = right after treatment (the endpoint with purpura is visible); C = after 2 treatments; D = multispectral melanin component, before and after the last treatment; E = multispectral vascular component, before and after the last treatment.

**FIGURE 12:**
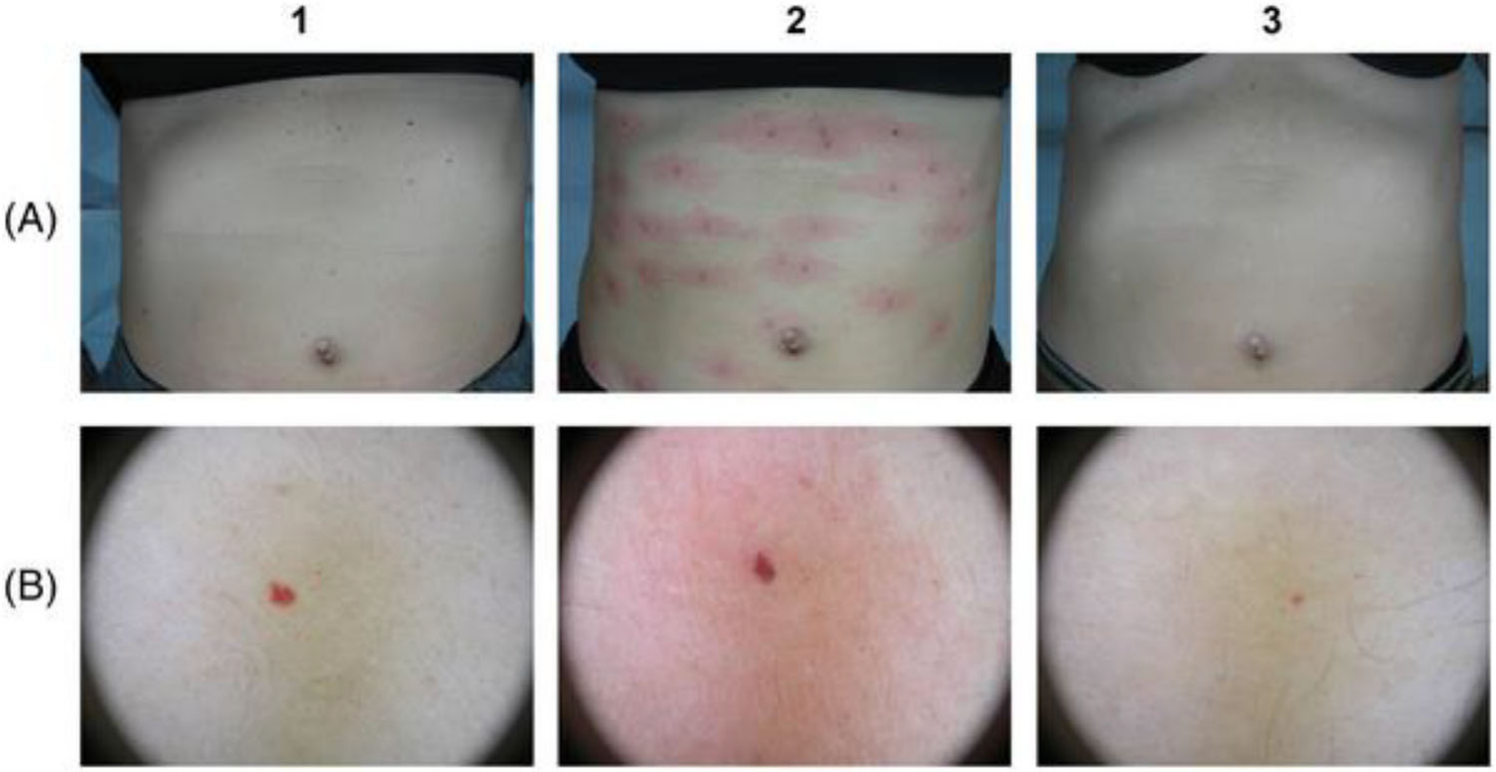
Clinical assessment of trunk cherry (ruby) angiomas (A) in a woman patient. The progression and a full resolution are visible also when the dermatoscopic analysis is performed (B) before (1), right after (2), and after (3) the last treatment (just 1).

